# Impairments in the prefronto-hippocampal interactions explain associative learning deficit in schizophrenia

**DOI:** 10.1186/1471-2202-12-S1-P93

**Published:** 2011-07-18

**Authors:** Mihály Bányai, Balázs Ujfalussy, Vaibhav Diwadkar, Péter Érdi

**Affiliations:** 1 KFKI Research Institute for Particle and Nuclear Physics, Hungarian Academy of Sciences, Budapest, Hungary; 2 Center for Complex Systems Studies, Kalamazoo College, Kalamazoo, MI, USA; 3 Wayne State University School of Medicine, Detroit, MI, USA

## 

Schizophrenia is a complex disorder that manifests on many different levels ranging from the physiology of single neurons thorough the dynamics of neural circuits to cognitive, affective or behavioral symptoms. We use a multi-level approach to connect these different levels. Our previous results demonstrated the schizophrenic patients show a significant impairment in object-location associative learning tasks (Diwadkar et al., 2008). Here we developed a neural network model incorporating brain regions involved in paired-associate learning in order to analyze the mechanisms underlying behavioral differences between schizophrenic patients and control subjects.

**Figure 1:**
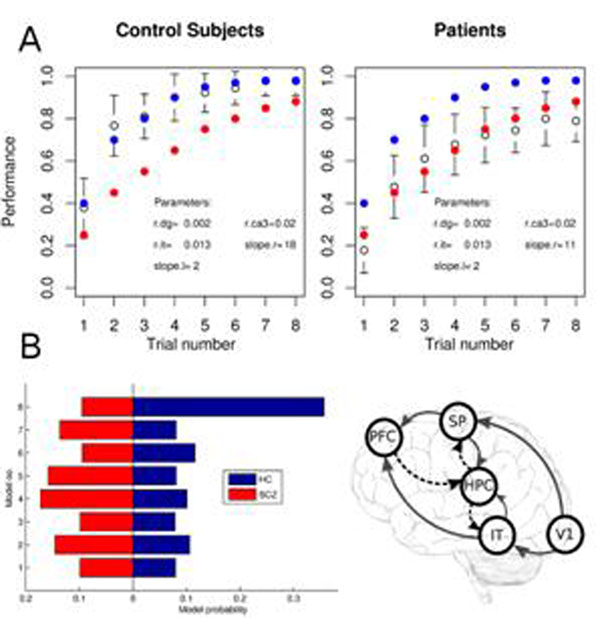
A. Learning curves of schizophrenia patients (red) healthy controls (blue) and simulation results from the neural network model (white) with two parameter settings. B. Model comparison results showing impairment in prefrontal control of memory formation in patients.

A feed-forward network creates the representations of the identity of the object and its location in the model of the area IT and SP in the ventral and dorsal visual stream, respectively. The proposed role of the hippocampus is to bind these two representations together so that when cued by the location, the correct object can be recalled. Moreover, in order to model cognitive control, we included a prefrontal region which controls learning and recall processes presumably by modulating the plasticity and the efficiency of hippocampal synapses. We fitted the model's performance to the behavioral data (Fig. 1A). Our model predicts that the impairment of cognitive control of the prefrontal cortex over hippocampal processes implies inaccurate regulation of hippocampal dynamics and explains the poorer performance of patients in this task.

Second, using a high-level probabilistic approach to model fMRI data recorded from the patients, we tested the predictions of the neural network model (Fig. 1B). We defined a set of generative models of the BOLD signal to describe the interaction of five brain regions included in the neural network model and the experimental conditions. The models were fitted to the data using Bayesian parameter estimation. The comparison of different model connectivity structures lead to the finding that in schizophrenia, the task-related functional network is fundamentally different relative to healthy controls, implying significant impairments in the prefrontal control of hippocampal memory formation in patients.

In conclusion, prefronto-hippocampal interactions are material in understanding learning impairments in schizophrenia, and our multi-level approach is suitable to integrate the explanatory capabilities of mechanistic neural models with the analytical power of data-driven phenomenological approaches.
